# Long-term in vivo vitamin D_3_ supplementation modulates bovine IL-1 and chemokine responses

**DOI:** 10.1038/s41598-023-37427-z

**Published:** 2023-07-05

**Authors:** Cian Reid, Susana Flores-Villalva, Aude Remot, Emer Kennedy, Cliona O’Farrelly, Kieran G. Meade

**Affiliations:** 1 Animal & Bioscience Research Department, Animal & Grassland Research and Innovation Centre, Teagasc, Grange, Co Meath Ireland; 2 School of Biochemistry and Immunology, Trinity College Dublin, Dublin 2, Ireland; 3 School of Agriculture and Food Science, University College Dublin, Belfield, Dublin 4, Ireland; 4 CENID Salud Animal e Inocuidad, INIFAP, Mexico, Mexico; 5 INRAE, Université de Tours, ISP, Nouzilly, France; 6 Teagasc, Animal & Grassland Research and Innovation Centre, Moorepark, Fermoy, Co. Cork Ireland; 7 School of Medicine, Trinity College Dublin, Dublin 2, Ireland; 8 Conway Institute of Biomolecular and Biomedical Research, University College Dublin, Belfield, Dublin 4, Ireland; 9 Institute of Food and Health, University College Dublin, Belfield, Dublin 4, Ireland

**Keywords:** Chemokines, Innate immunity

## Abstract

Vitamin D deficiency at birth, followed by prolonged insufficiency in early life may predispose bovine calves to infection and disease. However, the effects of vitamin D levels on innate immunity are unclear due to the lack of long-term supplementation trials in vivo and reliable approaches for reproducibly assessing immune function. Here, a standardized whole blood immunophenotyping assay was used to compare innate immune responses to infection relevant ligands (LPS, Pam3CSK4 and R848) between Holstein–Friesian calves supplemented with vitamin D (n = 12) from birth until 7 months of age and control calves (n = 10) raised on an industry standard diet. Transcriptomic analysis in unstimulated whole blood cells revealed increased expression of type I interferons and chemokines in vitamin D supplemented calves, while IL-1 and inflammasome gene expression was decreased. In response to stimulation with the bacterial ligand LPS, supplemented calves had significantly increased expression of *CASP1*, *CX3CR1*, *CAT*, whereas *STAT1* was decreased*.* Stimulation with the bacterial ligand Pam3CSK4 revealed increased expression of *IL1A*, *IL1B* and *CAT* genes; and decreased *C5AR1* expression. In response to the viral ligand R848, *STAT1* and *S100A8* expression was significantly decreased. An increased IL-1 and inflammasome gene expression signature in vitamin D supplemented calves in response to LPS and Pam3CSK4 was also found, with ELISA confirming increased IL-1β protein production. In contrast, a decreased chemokine gene expression signature was found in response to R848 in supplemented animals, with decreased IL-8 protein expression exhibited in response to all PAMPs also found. These results demonstrated expression of several cytokine, chemokine and inflammasome genes were impacted by vitamin D supplementation in the first 7 months of life, with IL-8 expression particularly responsive to vitamin D. Overall, vitamin D supplementation induced differential innate immune responses of blood immune cells that could have important implications for disease susceptibility in cattle.

## Introduction

Vitamin D is a hormone which has a well-defined role in calcium homeostasis and bone development. Many studies have also elucidated a significant impact of vitamin D on the human immune system and protection against disease^1,2^, while other studies did not demonstrate a significant relationship^3,4^. However, the possibility that vitamin D supplementation might improve bovine resistance to immune mediated disease such as chronic inflammation and also infection could have significant economic and welfare implications. Controlled supplementation trials in animals may also contribute to defining effects of vitamin D on immune functions relevant for human health.

Vitamin D enters the body through diet or via sunlight exposure and is converted to inactive 25(OH)D in the liver. It is transported through the blood by vitamin D binding protein (DBP) and converted to its active form (125(OH)_2_D) through hydrolysis by the 1α-hydroxylase enzyme, encoded by the *CYP27B1* gene, in the kidney. 125(OH)_2_D bound to DBP circulates throughout the body where it can enter multiple cell types through endocytosis or diffusion. Within the cell it binds to the vitamin D receptor (VDR) and heterodimerizes with the retinoid X receptor. This nuclear complex translocates to the nucleus where it can bind to DNA at vitamin D response elements (VDRE) sites in gene promoter regions to regulate transcription of genes involved in calcium absorption, a key process in bone development. *CYP27B1* and *VDR* genes are widely expressed across cell types, and thereby have the capacity to produce active vitamin D and effect transcription of genes involved in many physiological processes, in particular immunity, where conversion of vitamin D to its active form is increased in immune cells upon stimulation^5^.

Vitamin D status is determined through measuring 25(OH)D in serum by protein quantification techniques i.e. ELISA. 25(OH)D is a stable form of vitamin D, with a half-life of weeks making it a more accurate reflection of vitamin D status over time than other metabolites^6^. Vitamin D deficiency is defined as less than 20 ng/mL of 25(OH)D in serum^7,8^ and in humans, vitamin D deficiency is associated with increased susceptibility to respiratory tract infections^9^ including tuberculosis (TB)^10^, COVID-19^11^ and multiple other inflammatory diseases^12^. In cattle, low 25(OH)D is associated with Johne’s disease (also known as paratuberculosis)^13^ as well as TB^14^. Deficiency and prolonged insufficiency (20–30 ng/mL) have been documented in the early life of calves^15^ which is a period of high disease incidence, morbidity and mortality.

Vitamin D supplementation has been shown to have a role in preventing acute respiratory infections in humans^16^ and in reducing the severity of COVID-19^17^. In cattle, vitamin D supplementation decreased the incidence of metritis^18^ and therefore, vitamin D supplementation may offer an opportunity to reduce the disease burden and mortality in calves. A mechanism by which vitamin D may improve disease resistance is through modulation of innate immunity. The vitamin D pathway activates cathelicidin host defense peptides in human macrophages upon TLR stimulation^5^. The same study demonstrated an association between low 25(OH)D and cathelicidin expression in African Americans (who have increased TB susceptibility). Vitamin D has been shown in humans to modulate cytokine responses to bacterial ligands via increased IL-10 and decreased IL-1, IL-6, IL-8 and TNF-α expression in multiple cell types in vitro, including monocytes, macrophages and PBMCs^19,20^. Vitamin D also had suppressive effects on chemokine expression in *Mycobacterium tuberculosis* stimulated PBMCs from patients with TB in vitro^21^. Therefore, it is postulated that vitamin D may be protective against disease via modulation of bactericidal and inflammatory cytokine activity.

In vitro studies have limited ability to capture the complex effects of vitamin D throughout the body, which involves hydroxylation in both the liver and the kidney as well as in individual immune cells. Characterization of the effects of vitamin D supplementation on innate immunity are also challenging due to the lack of long-term in vivo supplementation trials, particularly in cattle. Using an in vivo model, we have previously reported significantly altered cellular immunity in bovine calves supplemented with vitamin D from birth to 7 months of age through decreased circulating granulocytes^22^. The objective of this study was to determine the molecular changes in immune status and responses to pathogen associated molecular patterns (PAMPs) by blood immune cells from these same vitamin D supplemented animals. Using ImmunoChek, our previously optimised technique for analyzing innate immune activity^23^, we stimulated blood cells with PAMPs that mimic bacterial and viral immune stimulation through Toll-like receptor (TLR) activation. We measured gene expression via multiplex qPCR technique Fluidigm Biomark after 7 months of vitamin D supplementation, which was the last timepoint before cessation of vitamin D supplementation and therefore after maximum vitamin D exposure in this trial. We focused on genes important for innate cytokine signaling, inflammatory pathways and molecules which mediate immune responses against bacterial and viral infection. Protein expression of innate cytokines IL-1β, IL-6 and IL-8 were measured by ELISA longitudinally after 3, 5 and 7 months of vitamin D supplementation.

## Results and discussion

### Vitamin D supplementation elevates type I interferon, chemokine and PRR gene expression and decreases IL-1 and inflammasome gene expression in unstimulated samples

**Figure 1:**
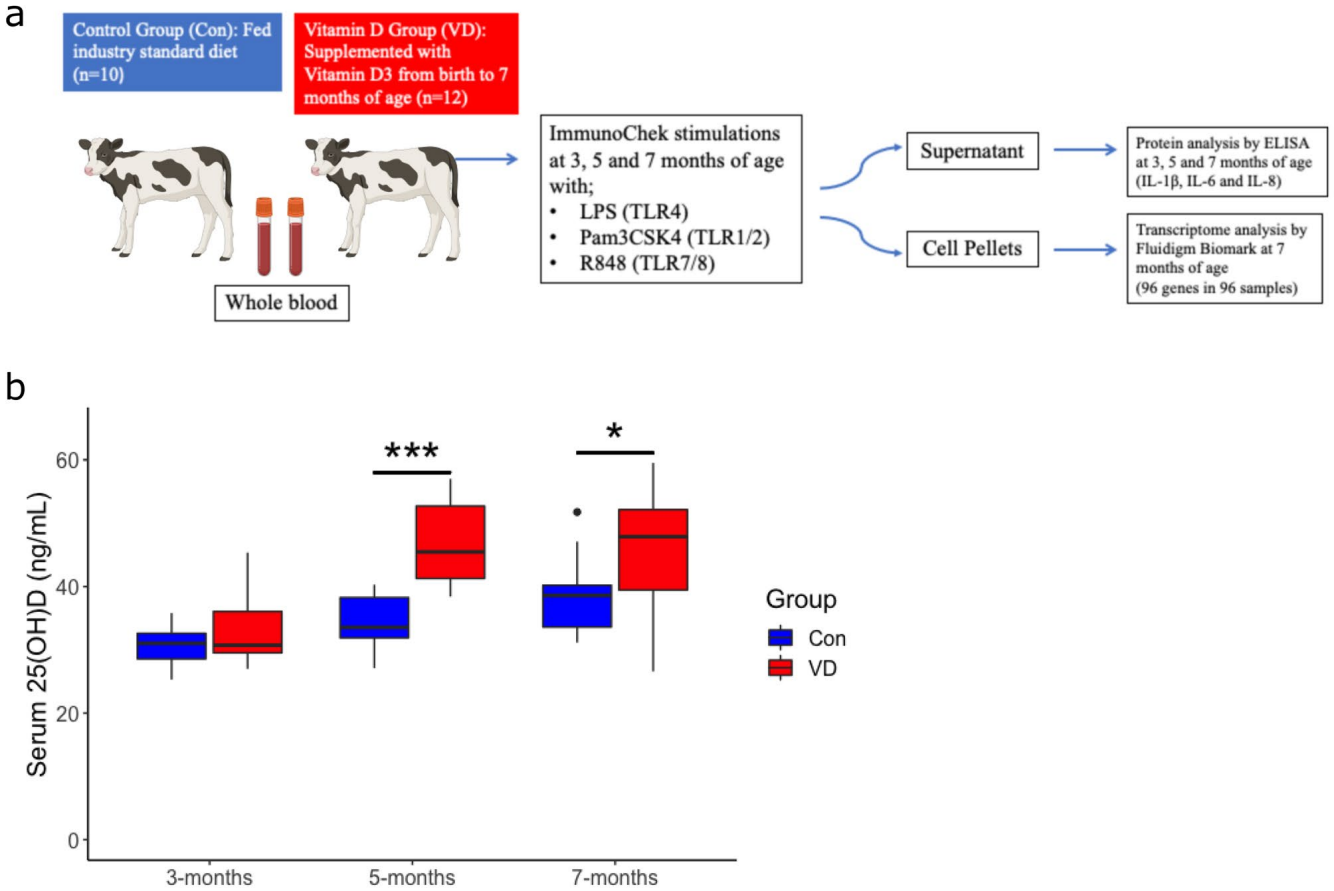
Experimental design. (**a**) 10 calves were fed industry diet (Con) while 12 calves were supplemented with vitamin D3 from birth to 7 months of age (VD), all male Holstein Friesians, as per *Flores-Villalva *et al.^22^. Ex-vivo whole blood stimulations were carried out in which whole blood collected at 3, 5, 7 months of age was stimulated with null (unstimulated/baseline) and disease relevant PAMPs (LPS, Pam3CSK4 and R848). Protein analysis (IL-1β, IL-6, IL-8) was carried out by ELISA at 3, 5 and 7 months of age in the supernatant of the stimulations and transcriptome analysis was carried out using Fluidigm Biomark at 7 months in RNA extracted from the cell pellets (created in Biorender.com). (**b**) 25(OH)D concentrations in serum of control and vitamin D supplemented calves at 3, 5 and 7 months age, as per *Flores-Villalva *et al.^22^; *p < 0.05, **p < 0.01, ***p < 0.001.

**Figure 2:**
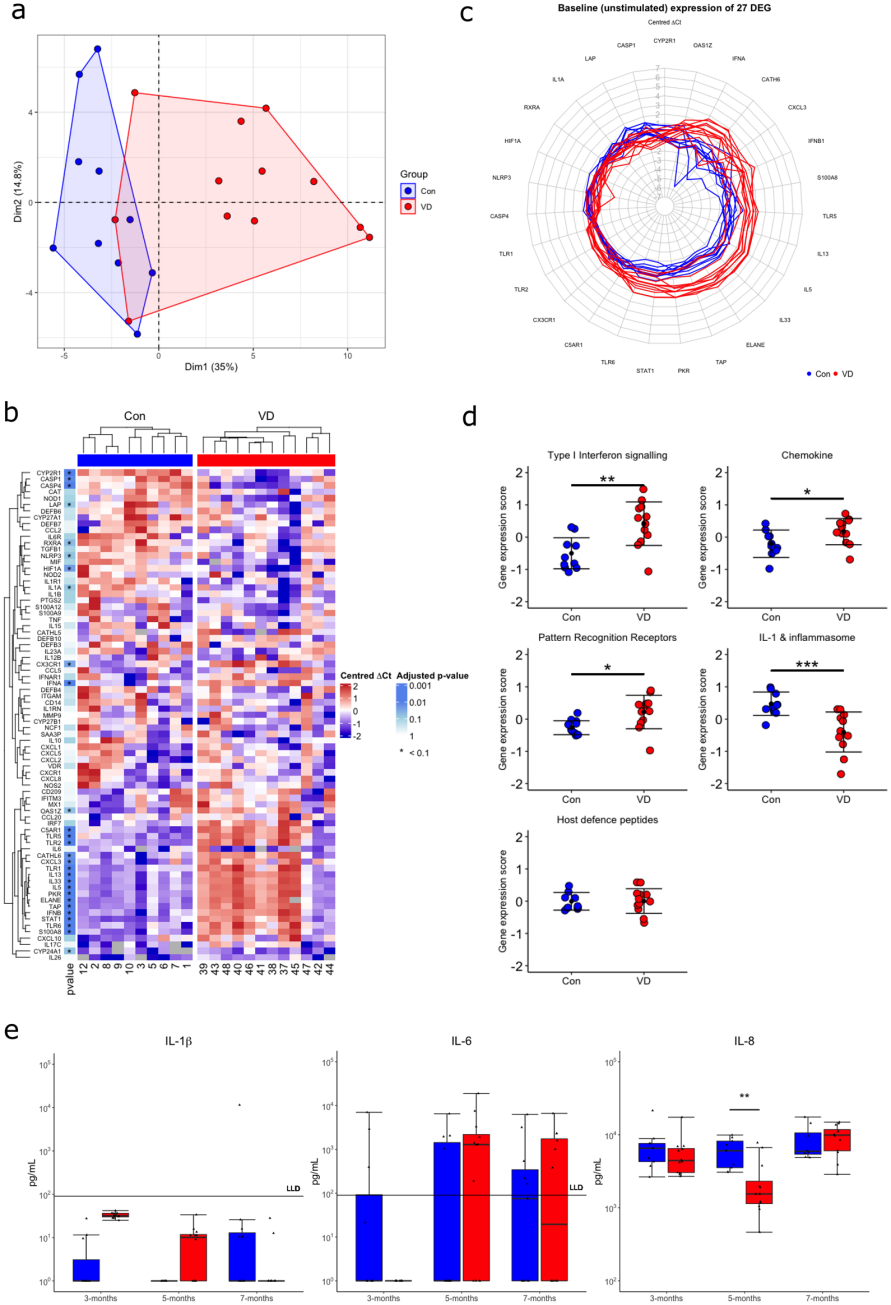
Vitamin D supplemented calves (VD) exhibit 27 differentially expressed genes in comparison to controls in unstimulated whole blood (Con). (**a**) PCA analysis of unstimulated gene expression of 77 genes in Con and VD calves. (**b**) Heatmap of relative unstimulated expression (mean centred dCt) of 77 genes in Con and VD calves and p value results of multiple *t* tests after p value adjustment: *p < 0.1. (**c**) Radar plot of the relative expression (mean centred dCt) of the 27 genes found differentially expressed between Con and VD calves. (**d**) Unstimulated gene expression z-scores of gene groups in Con and VD calves; *p < 0.05, **p < 0.01, ***p < 0.001. (**e**) Protein expression of IL-1β, IL-6 and IL-8 in unstimulated samples supernatant at 3, 5 and 7 months of age in Con and VD calves, with lower limit of detection (LLD) of ELISA labelled for IL-1β and IL-6; *p < 0.05, **p < 0.01.

Whole blood from control calves and calves that had been supplemented with vitamin D_3_ from birth was stimulated for 24 h with PAMPs LPS, Pam3CSK4 and R848 at 3, 5 and 7 months of age, as well as a 24h unstimulated condition (Fig. 1a). The vitamin D supplemented calves had significantly increased 25(OH)D concentrations at 5 and 7 months of age (Fig. 1b). Firstly, expression of 96 genes were measured in the unstimulated samples at 7 months of age. Sixteen genes exhibited no or low expression, and therefore the other 77 genes (excluding the normaliser genes) were used for subsequent analysis. Principal component analysis (PCA) of unstimulated (baseline) gene expression data from the 77 genes revealed that vitamin D supplemented calves clustered separately to controls, indicative of a distinct expression profile (Fig. 2a). Furthermore, hierarchical clustering revealed distinct baseline gene expression patterns between controls and supplemented calves, with 27 differentially expressed genes found after *p* value correction (Fig. 2b). This demonstrates changes in baseline immune status in supplemented calves. Nineteen of these genes were significantly increased (*TLR1, TLR2, TLR5, TLR6, IFNA, IFNB1, STAT1, OAS1Z, CATHL6, S100A8, TAP, CXCL3, CXC3CR1, IL13, IL5, IL33, ELANE, PKR* and *C5AR1*) and 8 genes were significantly decreased (*IL1A, CASP4, CASP1, NLRP3, CYP2R1, RXRA, HIF1A* and *LAP*) in the vitamin D supplemented group (Fig. 2c). This shows that vitamin D supplementation results in increased expression of one group of genes, while increasing regulation of another distinct group. Specifically, gene expression of type I interferons (*IFNA* and *IFNB1)*, interferon stimulated genes (*STAT1* and *OAS1Z),* chemokines (*CXCL3* and *CXC3CR1),* pattern recognition receptors (*TLR1, TLR2, TLR5* and *TLR6)*, host defense peptides (*CATHL6* and *TAP)* and Th2 helper-cell related cytokines (*IL13, IL5* and *IL33)* were increased in vitamin D supplemented animals, while *IL1A* and inflammasome genes (*CASP4, CASP1* and *NLRP3)* were decreased (Fig. 2c). Furthermore, significantly increased type 1 interferon signaling, chemokine and PRR gene expression scores were found in unstimulated samples of supplemented animals, as well as decreased IL-1 and inflammasome gene expression scores (Fig. 2d). This further indicates that vitamin D is increasing type I IFN interferon signaling, chemokine and PRR gene expression at baseline, while also increasing regulation of IL-1 and inflammasome gene expression. No differences were found between host defense peptide gene expression scores (Fig. 2d).

Elevated type I IFN signaling gene expression has been shown in blood immune cells after supplementation in healthy humans, suggesting vitamin D enhances constitutive activity of this pathway, which is important for maintaining homeostasis and priming immune cells for improved vaccine responses^24–26^. Similarly, increased PRR expression by vitamin D has previously been demonstrated in healthy humans on diets supplemented with vitamin D which may protect against disease through increased immune surveillance^27^. Vitamin D supplementation has previously been found to increase chemokine gene expression in cattle^28^. Vitamin D has been shown to decrease expression of IL-1 and inflammasome genes in vitro, perhap*s* protecting against pathological inflammation^29^. While overall host defense peptide gene expression z-scores were similar between groups, significant increases in *CATHL6* and *TAP* were apparent. These antimicrobial peptides may confer increased disease protection as they have potent anti-microbial potential and are important in the immune response to bovine respiratory disease, endometritis and mastitis^30–32^. Expression of *IL13, IL5* and *IL33* were also increased by vitamin D supplementation and may contribute to increased Th2 helper cell activation, which vitamin D has been previously shown to induce in mice, providing protection against several autoimmune diseases^33–36^.

IL-1β, IL-6 and IL-8 protein concentrations were measured by ELISA in the supernatant of unstimulated samples at 3, 5 and 7 months of age. IL-8 protein concentrations were significantly decreased after 5 months of vitamin D supplementation in unstimulated samples (Fig. 2e). IL-8 has a key role in the recruitment of neutrophils, T cells and other immune populations, suggesting decreased trafficking of these cell types in supplemented calves. Previous work in cattle identified an inverse correlation between IL-8 protein expression and circulating 25(OH)D concentrations^15^. Concentrations of IL-1β and IL-6 were consistently below the lower limit of detection (30 pg/mL).

Overall, our study shows that vitamin D supplementation changes the innate immune status of calves through elevated type I IFN signaling, chemokine and PRR gene expression and decreased IL-1 and inflammasome gene expression. Changes in immune status could have important implications for susceptibility to infection and inflammatory disease, as well as the response to vaccination^37,38^.

### PAMP-specific induced immune responses in stimulated whole blood from calves

**Figure 3:**
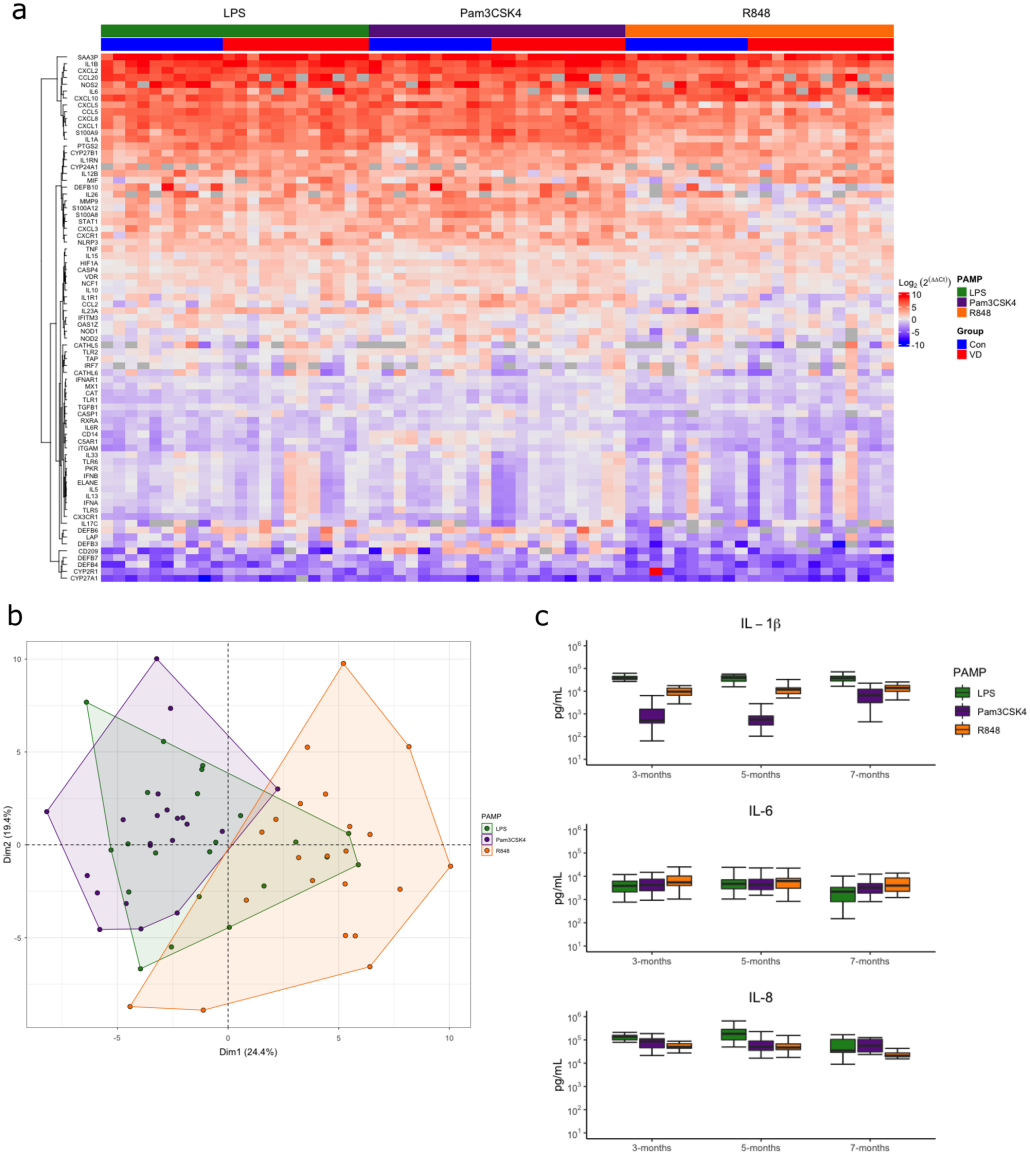
PAMP induced gene expression of immune related genes in 7-month-old control (Con) and vitamin D supplemented (VD) calves supplemented are PAMP specific. (**a**) PCA analysis PAMP induced fold changes of 77 genes in response relative to unstimulated expression. (**b**) Heatmap of PAMP induced fold changes of 77 genes relative to unstimulated expression. (**c**) Boxplots of protein expression of IL-1β, IL-6 and IL-8 in LPS, Pam3CSK4 and R848 stimulated samples supernatant at 3, 5 and 7 months of age in all calves.

Gene expression of 96 genes was measured in PAMP (LPS, Pam3CSK4 and R848) stimulated samples at 7 months of age and found sixteen genes exhibited no or low expression, and therefore the other 77 genes (excluding the 3 normaliser genes) were used for subsequent analysis. Hierarchical clustering of gene expression revealed a distinct bimodal response profile (Fig. 3a). Upregulation of multiple genes involved in the inflammatory response were detected in response to all three PAMPs, including cytokines, chemokines, inflammasome pathway and vitamin D signaling, relative to paired unstimulated whole blood samples (Fig. 3a). In contrast, multiple additional immune genes were found to be downregulated upon PAMP stimulation, including type I interferon signaling genes (Fig. 3a). R848 mediated downregulation of both type I IFN genes *IFNA* and *IFNB1* was unexpected as R848 is a viral ligand and successfully upregulated multiple inflammatory genes in this experiment, and has been found to upregulate type I IFN genes in human whole blood^39^. Additionally, type I IFN gene expression is increased in cattle infected with bovine viral diarrhea virus^40^. Possible reasons for this could be species-specific differences in type I interferon responses, or alternatively after 24 h of stimulation the interferon response to R848 had been resolved.

PCA analysis identified clustering of individual immune responses based on PAMP stimulation, where Pam3CSK4 and R848 induced responses clustered separately across PC1 and LPS induced responses clustered similarly to both Pam3CSK4 and R848 (Fig. 3b). A PCA variables plot was constructed to identify genes contributing to the differences seen across PC1 and therefore differences between Pam3CSK4 and R848 (Fig. S1). Increased expression of multiple inflammatory genes involved in IL-1 and inflammasome signaling, chemokine signaling and vitamin D signaling was found associated with Pam3CSK4 compared to R848, whereas increased *CXCL10* and *IL6* was found associated with R848 compared to Pam3CSK4 (Fig. S1). This shows that the induced immune responses are PAMP specific, and that responses to bacterial ligand Pam3CSK4 showed distinct patterns of gene expression compared to viral ligand R848.

IL-1β, IL-6 and IL-8 protein concentrations were measured by ELISA in the supernatant of PAMP stimulated samples at 3, 5 and 7 months of age. Increased IL-1β in response to LPS and R848 compared to Pam3CSK4 at 3, 5 and 7 months was found (Fig. 3c). As large a magnitude of difference between PAMPs in IL-6 and IL-8 was not observed compared to IL-1β (Fig. 3c). However, increased median values can be observed in IL-6 in response to R848, and in IL-8 in response to LPS at 3 and 5 months, compared to other PAMPs (Fig. 3c). This further highlights PAMP-specific cytokine responses but at a protein level.

This demonstrates successful immune activation using a standardized whole-blood PAMP stimulation assay and characterizes early age immune responses in calves to bacterial and viral PAMPs. Differences in cytokine responses to multiple TLR ligands in bovine whole blood stimulation assays has been previously characterized at a protein level^23,41^. However, this study identified different gene expression signatures associated with bacterial and viral ligands from 77 genes involved in the bovine immune response using a standardized assay. This has also been shown in humans using whole-blood stimulations along with gene expression analysis, where unique immune response gene signatures were found associated with multiple complex stimuli^39^. This highlights that defining healthy or disease associative innate immune responses will be dependent on the immune stimuli, or pathogen involved.

### Vitamin D supplemented calves have increased IL-1 and inflammasome and decreased chemokine gene expression in response to PAMPs

Next, comparative analysis of gene expression in response to PAMPs was carried out between control and vitamin D supplemented calves. PCA analysis of the gene expression of 77 genes in response to LPS (Fig. S2a), Pam3CSK4 (Fig. S2b) and R848 (Fig. S2c) relative to unstimulated cells did not result in a distinct clustering profile based on supplementation status. Additionally, hierarchical clustering analysis did not reveal distinct patterns of individual gene expression in response to any PAMP (Fig. S3a–c).

**Figure 4:**
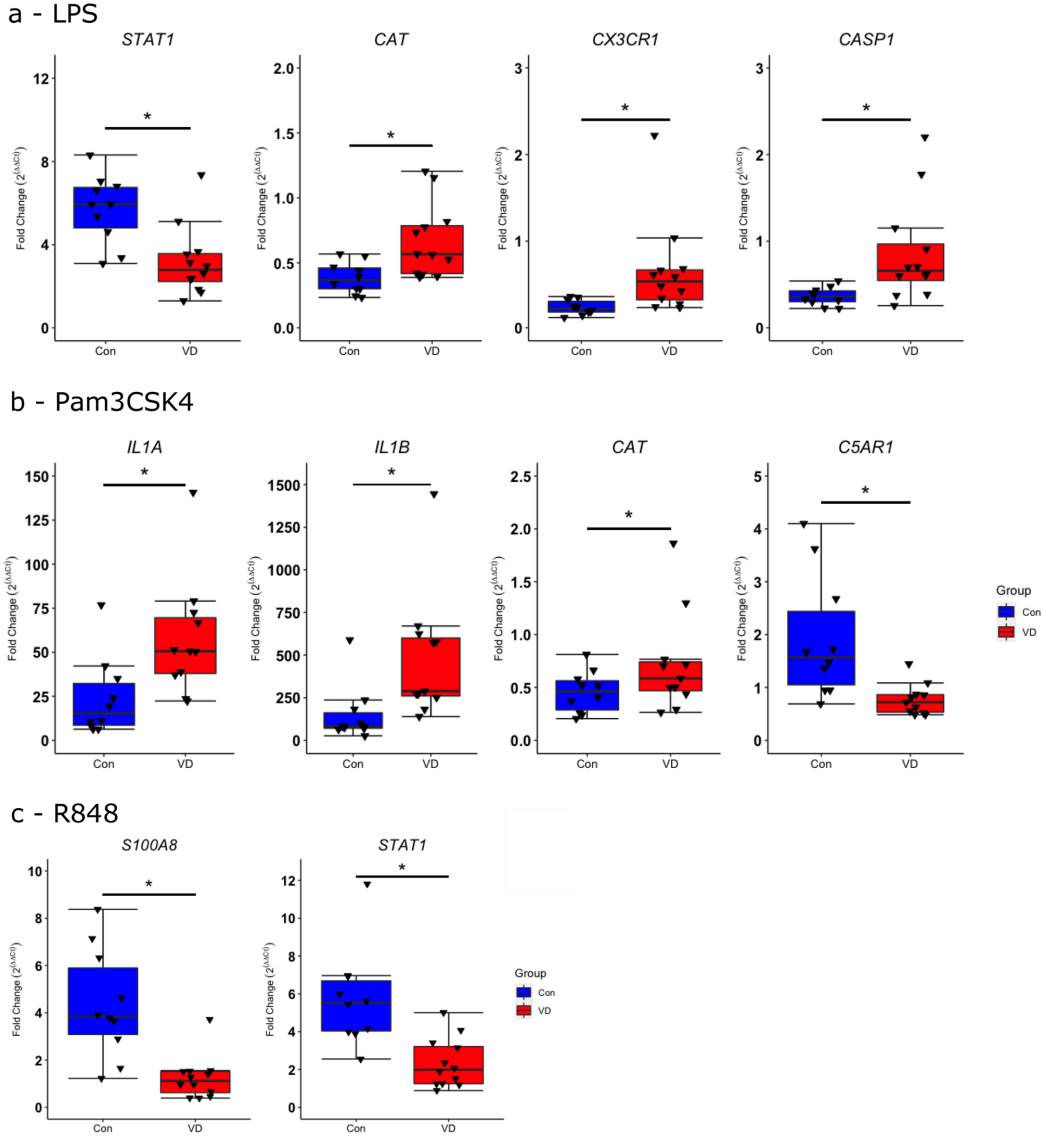
Significant differential expression in response to PAMPs between vitamin D supplemented (VD) and control (Con) calves. Fold change of significantly differentially expressed genes in response to (**a**) LPS (*STAT1, CAT, CX3CR1* and *CASP1),* (**b**) Pam3CSK4 (*IL1A, IL-1B, CAT* and *C5AR1)* and (**c**) R848 (*S100A8* and *STAT1),* post p value adjustment between Con and VD calves. *p adjusted < 0.1.

**Figure 5:**
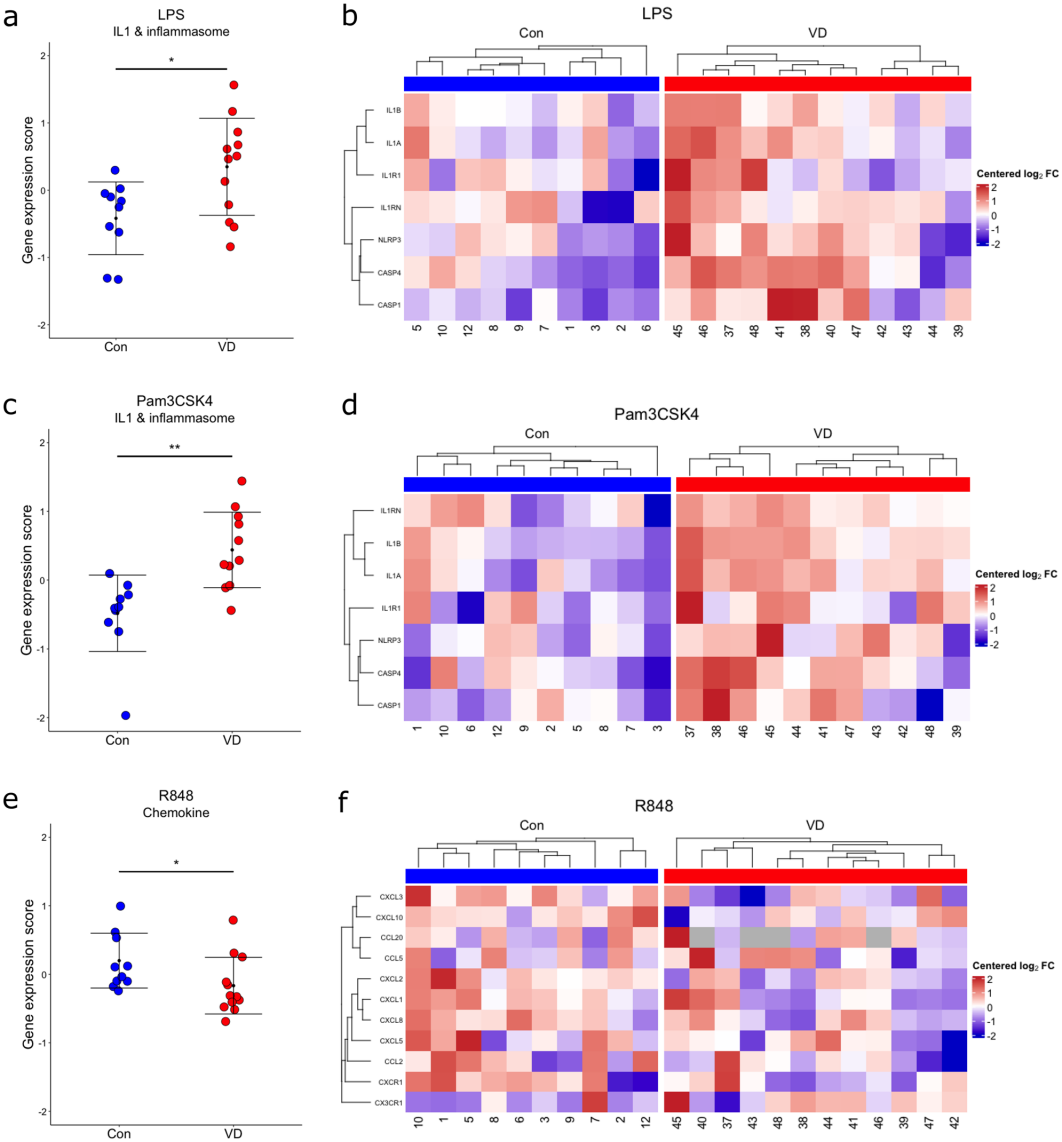
Vitamin D (VD) supplemented calves have increased expression of IL-1 and inflammasome genes in response to LPS and Pam3CSK4, and decreased expression chemokine genes in response to R848. Gene expression z-scores for IL-1 and inflammasome genes in response to (**a**) LPS, (**c**) Pam3CSK4 and chemokine gene expression in response to (**e**) R848 of Con and VD calves; *p < 0.05, **p < 0.01; Heatmap of differentially regulated IL-1 and inflammasome genes in response to (**b**) LPS and (**d**) Pam3CSK4 and chemokine genes in response to (**f**) R848 in Con and VD calves.

In response to LPS, expression of *CAT*, *CX3CR1* and *CASP1* genes were significantly increased and *STAT1* gene was significantly decreased in supplemented calves after *p* value adjustment (Fig. 4a). In response to Pam3CSK4, expression of *IL1A*, *IL1B* and *CAT* genes were similarly increased and *C5AR1* gene was significantly decreased in supplemented calves (Fig. 4b). In response to R848, expression of *S100A8* and *STAT1* genes were significantly decreased in supplemented calves (Fig. 4c). This demonstrates that vitamin D supplementation is regulating innate immune gene expression responses to PAMP stimulation. Furthermore, gene expression scores were calculated, and IL-1 and inflammasome gene expression scores were significantly increased in supplemented calves in response to LPS (Fig. 5a) and Pam3CSK4 (Fig. 5c), whereas chemokine gene expression scores were significantly decreased in response to R848 (Fig. 5e). A distinct increased expression pattern for the genes involved in IL-1 and inflammasome signaling (*IL1A, IL1B, IL1R1, IL1RN, NLRP3, CASP1, CASP4*) can be observed in response to LPS (Fig. 5b) and Pam3CSK4 (Fig. 5d), and a decreased expression pattern for chemokine genes can be observed in response to R848 (Fig. 5f). This shows vitamin D supplementation is modulating immune responses to PAMPs through increased IL-1 and inflammasome gene expression and decreased chemokine gene expression.

Previous literature has shown that vitamin D can drive *IL1B* transcription in *Mycobacterium tuberculosis* infected macrophages and also increased expression of pro-IL-1β transcripts in vitro^42,43^. In vivo supplementation trials in cattle have also demonstrated increased *IL1B*, *IL1R1* and *IL1RN* mRNA transcripts in blood immune cells of pre- and post-partum cattle^44^. Another supplementation trial also found increased *IL1B* transcripts in milk somatic cells^45^. Therefore, supplemented animals may have an enhanced inflammatory response through increased expression of IL1 gene family, as well as inflammasome complex genes to mediate IL-1β release from the cell. However, multiple in vitro studies have also elucidated decreased inflammasome activity attributed to vitamin D^46,47^. Differences between studies may also be due to PAMP or pathogen, cell type, or species being investigated. Furthermore, vitamin D has also been previously shown to suppress the chemokine response^21^. Interestingly, our study only shows a suppressive effect on chemokine gene expression in response to viral ligand R848. An in vitro study found that vitamin D decreased chemokine expression in response to respiratory syncytial virus while maintaining the same levels of viral clearance^48^. Therefore, vitamin D supplemented calves may have enhanced regulation of the chemokine gene expression upon immune stimulation, to prevent pathological inflammation.

Interestingly, chemokine gene expression scores were found elevated at baseline in supplemented calves but decreased in response to stimulation. Conversely, IL-1 and inflammasome gene expression scores were decreased at baseline but elevated in response to stimulation. This can also be seen for individual genes in the supplemented calves, with *STAT1* and *C5AR1* displaying increased baseline expression and decreased expression in response to stimulation, and *IL1A* and *CASP1* displaying decreased baseline expression and increased expression in response to stimulation. This identifies two types of vitamin D responsive gene subsets, where one gene subset has elevated expression at baseline line which in turn limits its immune response to PAMPs, and another which has decreased baseline expression, which in turn elevates it response to PAMPs. This shows through gene expression that vitamin D supplementation is changing how immune cells are primed which is affecting the immune response, possibly through regulation of epigenetic mechanisms^49^.

Overall, our study found vitamin D supplementation is modulating immune responses to PAMPs through an increased IL-1 and inflammasome gene expression and decreased chemokine gene expression, which could have important implications for susceptibility to infection and pathological inflammation.

### Increased IL-1β and decreased IL-8 protein in response to PAMPs in vitamin D supplemented calves

**Figure 6:**
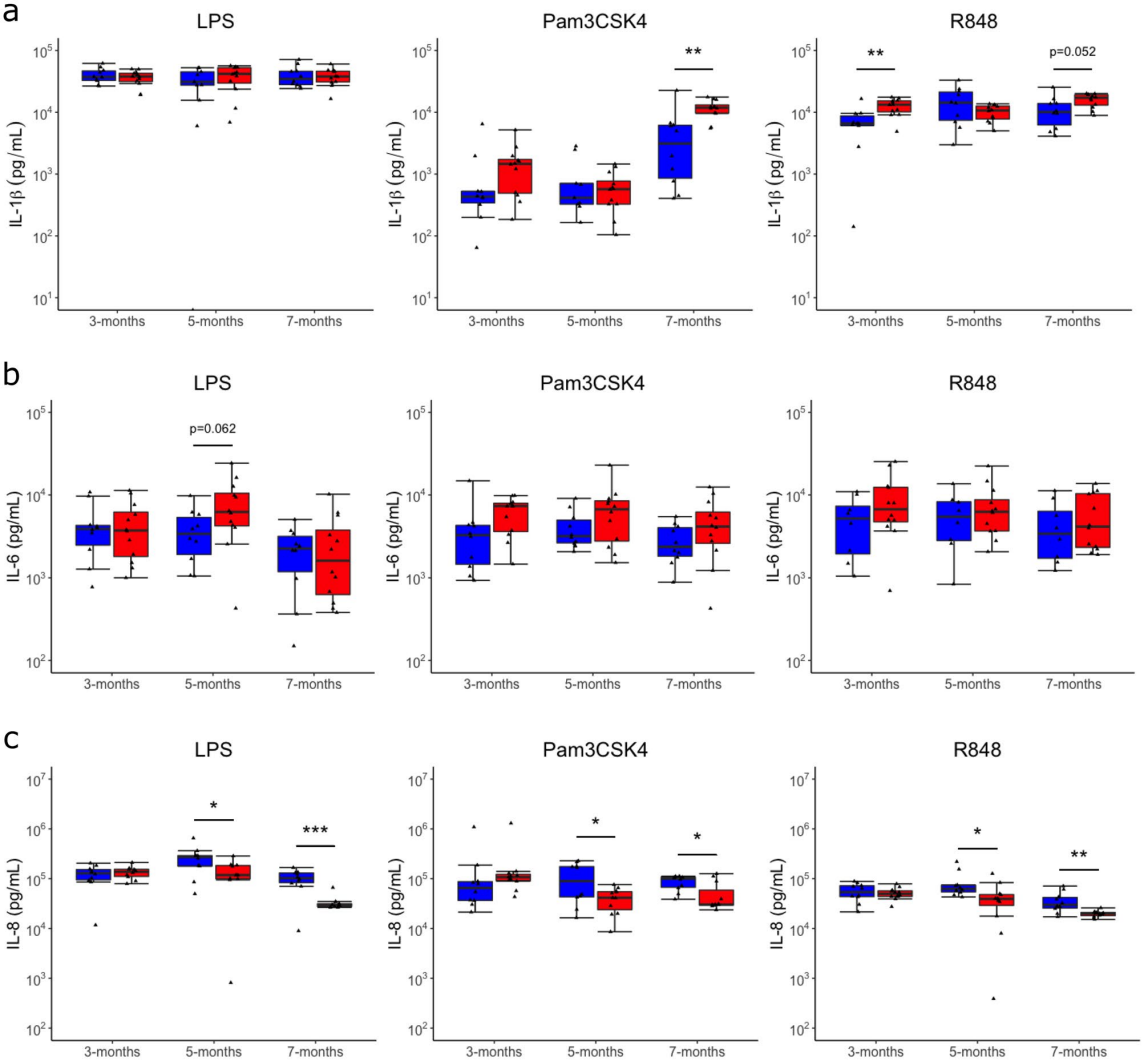
Vitamin D supplemented calves (VD) have increased IL-1β in response to Pam3CSK4 and R848 and decreased IL-8 in response to LPS, Pam3CSK4 and R848 in compared to control calves (Con). Boxplots of protein expression of (**a**) IL-1β, (**b**) IL-6 and (**c**) IL-8 in LPS, Pam3CSK4 and R848 stimulated samples supernatant at 3, 5 and 7 months of age in Con and VD calves; *p < 0.05, **p < 0.01, ***p < 0.001, ****p < 0.0001.

Finally, comparative analysis of protein expression of IL-1β, IL-6 and IL-8 in response to PAMPs was carried out between control and vitamin D supplemented calves. Protein expression of cytokine IL-1β was significantly increased in response to Pam3CSK4 at 7 months and in response to R848 and 3 months, in addition to close to significance (p = 0.052) at 7 months, in supplemented animals (Fig. 6a). No significant differences were found in response to LPS (Fig. 6a). This is consistent to what was found in gene expression, with increased IL-1β responses likely being due to increased IL-1 and inflammasome gene expression found as in vitro studies showed increased IL-1β protein release, due to vitamin D exposure, is dependent on NLRP3^29,42^. IL-1β has been shown to be elevated in multiple diseases in cattle and is a marker for development of pathological inflammation in endometritis^50–52^. However, in human studies, elevated IL-1β is linked to the release of anti-inflammatory cytokine IL-10 during inflammation, and hypoproduction of IL-1β was associated with inflammatory disease which results in increased disease severity^53–55^. It has also been shown to be protective during TB infection in mice^56^. Therefore, increased IL-1β responses driven by vitamin D supplementation may increase protective mechanisms against infection and inflammatory disease.

Protein expression of cytokine IL-6 was close to significantly increased in response to LPS at 5 months (p = 0.062) (Fig. 6b). No differences were found in response to Pam3CSK4 and R848 (Fig. 6b). Previous studies in humans have found contradictory evidence to this study, showing that vitamin D decreases IL-6 in individuals with pneumonia, indicating a possible role for vitamin D regulation of IL-6 responses in disease^57,58^.

Finally, protein expression of chemokine IL-8 was significantly decreased in response to LPS, Pam3CSK4 and R848 at 5 and 7 months of age. (Fig. 6c). These results are consistent with the transcriptomic data which showed reduced induced chemokine responses in supplemented animals. In vitro studies in humans have also shown decreased IL-8 protein responses due to vitamin D^59,60^. This indicates a potentially important role for vitamin D in the regulation of IL-8 in inflammation. Additionally, high IL-8 responses in bovine fibroblasts have been previously shown to be associated with increased tissue damage in mastitis, and therefore decreased IL-8 responses may reduce susceptibility to severe disease^61,62^.

Overall, our study shows vitamin D supplementation is modulating the immune response to PAMPs through increased IL-1β and decreased IL-8 protein expression, which is consistent with what was found in gene expression.

## Conclusion

This study involves the measurement of innate immune responses at transcriptomic and proteomic levels in calves supplemented with vitamin D throughout the first 7 months of life. Results show that vitamin D supplementation plays an important immunoregulatory role at baseline, increasing expression of immune genes such as type I interferons, chemokines, PPRs and decreasing IL-1 and inflammasome gene expression. Conversely, in response to PAMPs, supplementation enhanced IL-1 and inflammasome expression, while diminishing chemokine expression, with reductions in IL-8 protein exhibited in response to all PAMPs, showing particular sensitivity to supplementation. This adds to current evidence of the immunomodulatory effects of vitamin D supplementation to inflammatory pathways, and the changes in these pathways are likely to affect disease progression and outcomes in infected cattle.

## Materials and methods

### Animals

Holstein Friesian male calves were recruited from birth in the spring of 2020, and all housed at the Teagasc Animal and Bioscience Research Centre, Trim, Co. Meath for the duration of the study. Calves were maintained indoors in a standard agricultural environment with unrestricted access to food and water. Twelve calves were fed industry standard diet (6000 IU/kg in milk replacer + 2000 IU/kg of Vitamin D3 in ration) and twelve calves were given a vitamin D supplemented diet (single time injection of 50,000 IU of Vitamin D3 at birth, 10,000 IU/kg in milk replacer + 4000 IU/kg of Vit D3 in ration) from birth to 7 months old. Each time a new calf was recruited they were assigned to an alternate group as the previous to maintain a similar distribution of age between groups. Weight was measured upon arrival to the farm and similar distributions were found between groups. The mean ± standard deviation birth weight for controls and vitamin D supplemented animals were 42.0 ± 4.7 kg and 41 ± 5.7 kg respectively. The vitamin D3 supplemented milk replacer and ration (Rovimix D3 500, DSM Nutritional Products) was administered once a day at approximately 08:00 h. Two calves were removed from the control group as one died during the study, and the other was removed due to being an outlier in 31 of the genes measured in the unstimulated gene expression analysis as identified from Tukey analysis. The calves used in this study were part of a larger vitamin D supplementation trial. Reporting for the experiment was conducted in line with the *ARRIVE* guidelines and further details on vitamin D supplementation regime can be found in *Flores-Villalva *et al.^22^.

### Ex-vivo whole blood stimulations

Blood was collected from calves at 3, 5 and 7 months of age. The mean ± standard deviation age for controls and vitamin D supplemented animals were 72.3 ± 9.4 days and 70.9 ± 11.2 days at the 3-month timepoint, 127.3 ± 9.4 days and 125.9 ± 11.2 days at the 5-month timepoint, 196.3 ± 9.4 days and 194.9 ± 11.2 days at the 7-month timepoint respectively. At each timepoint, whole blood was collected via the jugular vein in 9 mL sodium heparin vacutainer in the morning (0800–1200 h) from each calf. 1 mL of whole blood from the calves was added to each of the following prefilled culture tubes (Sarstedt); 2mLs of media (unstimulated), 2ug/mL of LPS (Serotype O111:B4, Merck) in 2mLs of media, 1ug/mL of Pam3CSK4 (Invivogen) in 2mLs of media and 0.2ug/mL of R848 (Invivogen) in 2mLs of media. Media was prepared using RPMI media supplemented with 50 µg/mL streptomycin and 2.5 µg/mL amphotericin B (ThermoFisher Scientific). Following addition of whole blood, culture tubes were incubated at 38.5 °C for 24 h. Post incubation samples were centrifuged at 600×*g* for 10 min and supernatants were collected and stored at − 20 °C. Cell pellets were snap frozen in dry ice and stored at − 80 °C.

### RNA extraction, cDNA synthesis and gene expression analysis

RNA extraction was carried out using a combination of the Trizol method and the RNeasy Mini Kit from Qiagen^63^. Cell pellets from the stimulations carried out at 7 months were defrosted in 800 uL of trizol. Chloroform was added, the tubes were shaken vigorously and then centrifuged 12,000×*g* for 15 min. The solution was then transferred to a 2 mL microfuge tube and the same amount of 70% ethanol as sample was added. This was then added to the RNeasy column in which the rest of the procedure was carried out as per manufacturer instructions. RNA quantity was calculated using a nanodrop and RNA quality was calculated using a RNA Nano kit (Agilent Technologies) and a bioanalyzer. The collected RNA was then diluted to same concentrations and converted to cDNA using the High-Capacity cDNA Reverse Transcriptase kit, as per manufacturer instructions (Thermofisher). Gene expression analysis was carried out with cDNA using Biomark HD (Fluidigm) in a 96 × 96 well plate, as per manufacturer instructions. Fluidigm Real-Time qPCR software was used to calculate the Ct values for each gene in each sample. Primers used are shown in Supplementary Table 1. Ct values were then normalized to the two (*PPIA* and *ACTB*) housekeeping genes to calculate dCt values. Three normaliser genes were originally measured but *GAPDH* was removed due to unstable expression. Full details on the data generated using qPCR is shown in Supplementary Table 2. For the unstimulated expression, these values were centered to the mean and scaled by unit variance ((mean-dCt)/standard deviation) for use in PCA, heatmaps and radar plots. For PAMP induced responses, fold changes were calculated by normalizing the unstimulated expression using the deltadeltaCt method. The fold changes were then logged (log_2_(FC)) and mean centered and scaled by unit variance ((x-Log2FC)/standard deviation) for use in PCA and heatmaps.

### Gene expression scores

Gene expression scores were calculated using a z scoring system and has been previously used on gene expression results from *ex-vivo* blood stimulations in humans^64^. Firstly, genes were organized into groups based on signaling pathways and function. Z scores were then calculated for each gene by mean centering and scaling by unit variance, using the dCt value for unstimulated expression ((mean-dCt)/standard deviation), and Log2FC for PAMP stimulated expression ((x-Log2FC)/standard deviation). The average z score for each gene within a group was then calculated, giving each calf a gene expression score for that group.

### ELISAs

Protein analysis in the supernatants of the *ex-vivo* whole blood stimulations was carried out by using ELISAs IL-1 beta Bovine Uncoated ELISA Kit (Thermofisher) was used to measure IL-1β concentrations and IL-6 Bovine Uncoated ELISA kit (Thermofisher) was used to measure IL-6 concentrations, as per manufacturer instructions. IL-8 was measured as previously described^65^. Vitamin D levels were measured using a Human 25(OH)D ELISA (Eagle biosciences) with bovine standards, as previously described^66^.

### Statistical analysis

Statistical analysis was carried out and plots were made in R studio. Student t-tests were carried out to test differences between groups for gene expression. False discovery rate correction (Benjamini-Hochberg) was used for *p* value adjustment, with a significance cut off of *p* < 0.1. Unpaired *t* tests were used to calculate differences between groups for gene expression scores. Two-way ANOVA was used to calculate differences between groups in protein levels over 3 time points using weight difference between timepoints as a covariate. All box and dot plots were made with ggplot2 package, PCA plots in Factoextra package, heatmaps with ComplexHeatmaps package and radar plots with fmsb package. ComplexHeatmaps package carries out hierarchical clustering using the k-means clustering method.

### Ethical statement

All experimental procedures were approved by the Teagasc Ethics Committee (TAEC237-2019) and were conducted under the experimental license (AE19132/P105) from the Health Products Regulatory Authority in accordance with the cruelty to Animals Act (Ireland 1876) and the European Community Directive 2010/63/EU. Reporting in the manuscript follows the recommendations in the ARRIVE guidelines.

## Supplementary Information

Supplementary Figures.

Supplementary Table 1.

Supplementary Table 2.

## Data Availability

The datasets analysed during the current study are available from the corresponding author on reasonable request.
