# Supplementary material for: Long-term in vivo vitamin D3 supplementation modulates bovine IL-1 and chemokine responses
Source: Sci Rep. 2023 Jul 5;13:10846. doi: 10.1038/s41598-023-37427-z (PMC10322979; doi:10.1038/s41598-023-37427-z)
Supplement: Supplementary file 1 — Supplementary Figures. [file 41598_2023_37427_MOESM1_ESM.docx]

**Long-term *in vivo* vitamin D_3_ supplementation modulates bovine IL-1 and chemokine responses.**

Cian Reid Susana Flores-Villalva, Aude Remot, Emer Kennedy, Cliona O’ Farrelly and Kieran G. Meade

**Supplementary Figures**

**Supplementary Figure 1:** A PCA-biplot of the gene expression of 77 genes in unstimulated samples.

**Supplementary Figure 2:** PCA analysis of 77 genes in response (a) LPS, (b) Pam3CSK4 and (c) R848 in control (Con) and Vitamin D supplemented animals.

**Supplementary Figure 3:** Heatmap of 77 genes in response to (a) LPS, (b) Pam3CSK4 and (c) R848 in control (Con) and Vitamin D supplemented animals.

Figure S1


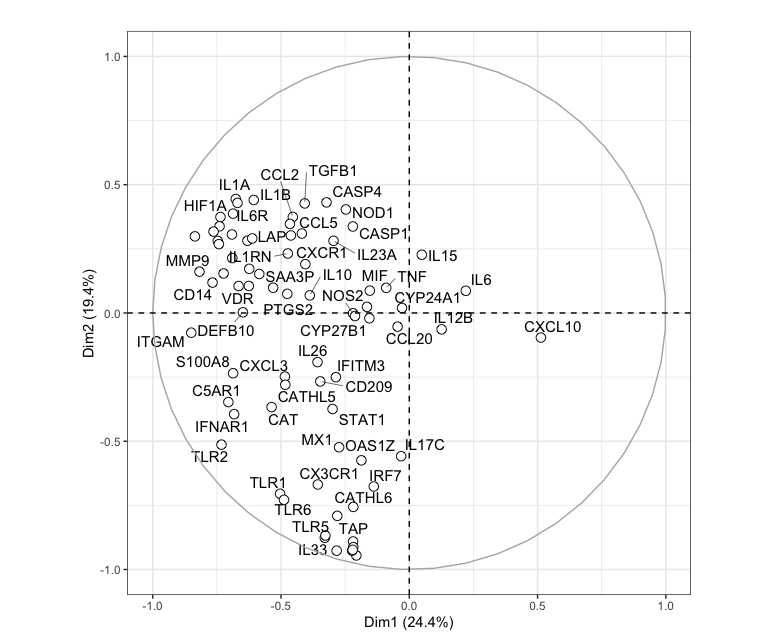


Figure S2

Figure S3
